# Formulation of pH-responsive highly swellable hydrogel scaffolds for controlled release of tramadol HCl: characterization and biocompatibility evaluation

**DOI:** 10.3389/fbioe.2023.1190322

**Published:** 2023-05-26

**Authors:** Zainab Abdullah, Muhammad Umer Ashraf, Kashif Barkat, Syed Faisal Badshah, Umaira Rehman, Asma Razzaq, Asif Mahmood, Farid Ulhaq, Hitesh Chopra, Summya Rashid, Marian Valko, Suliman Alomar, Kamil Kuca, Rohit Sharma

**Affiliations:** ^1^ Faculty of Pharmacy, University of Lahore, Lahore, Pakistan; ^2^ College of Pharmacy, University of Sargodha, Sargodha, Pakistan; ^3^ Department of Pharmacy, University of Chakwal, Chakwal, Pakistan; ^4^ Department of Chemistry, Division of Science and Technology, University of Education, Lahore, Punjab, Pakistan; ^5^ Chitkara College of Pharmacy, Chitkara University, Rajpura, Punjab, India; ^6^ Department of Pharmacology and Toxicology, College of Pharmacy, Prince Sattam Bin Abdulaziz University, Al-Kharj, Saudi Arabia; ^7^ Institute of Physical Chemistry and Chemical Physics, Faculty of Chemical and Food Technology, Slovak University of Technology, Bratislava, Slovakia; ^8^ Department of Zoology, College of Science, King Saud University, Riyadh, Saudi Arabia; ^9^ Department of Chemistry, Faculty of Science, University of Hradec Kralove, Hradec Kralove, Czechia; ^10^ Andalusian Research Institute in Data Science and Computational Intelligence (DaSCI), University of Granada, Granada, Spain; ^11^ Department of Rasa Shastra and Bhaishajya Kalpana, Faculty of Ayurveda, Institute of Medical Sciences, Banaras Hindu University, Varanasi, India

**Keywords:** controlled delivery, polymeric, pH responsive, tramadol HCL, controlled drug delivery, toxicity studies

## Abstract

**Introduction:** The objective of current project was to formulate a system for controlled delivery of Tramadol HCl (TRD), an opioid analgesic used in the treatment of moderate to severe pain.

**Methods:** For this purpose, a pH responsive AvT-co-poly hydrogel network was formulated through free radical polymerization by incorporating natural polymers i.e., aloe vera gel and tamarind gum, monomer and crosslinker. Formulated hydrogels were loaded with Tramadol HCl (TRD) and evaluated for percent drug loading, sol-gel fraction, dynamic and equilibrium swelling, morphological characteristics, structural features and in-vitro release of Tramadol HCl.

**Results and Discussions:** Hydrogels were proved to be pH sensitive as remarkable dynamic swelling response ranging within 2.94g/g-10.81g/g was noticed at pH 7.4 as compared to pH 1.2. Percent drug loading was in the range of 70.28%-90.64% for all formulations. Thermal stability and compatibility of hydrogel components were validated by DSC analysis and FTIR spectroscopy. Controlled release pattern of Tramadol HCl from the polymeric network was confirmed as maximum release of 92.22% was observed for over a period of 24 hours at pH 7.4. Moreover, oral toxicity studies were also conducted in rabbits to investigate the safety of hydrogels. No evidence of any toxicity, lesions and degeneration was reported, confirming the biocompatibility and safety of grafted system.

## 1 Introduction

Over the past few years, most researchers have taken an interest in developing novel drug delivery systems. At present, many researchers are prioritizing the development of dosage forms by using natural biomaterials since they are less toxic and biocompatible. Novel drug delivery systems not only provide enhanced patient compliance, which is achieved by decreasing the dosing frequency, but also enhance the therapeutic efficacy of active ingredients by minimizing fluctuations in the plasma level (Brahmareddy et al., 2015). Many pH-responsive drug delivery systems have also been fabricated. Delivery systems such as cationic liposomes, comprised of hydrophilic and hydrophobic moieties such as hydrocarbon chains or a cholesterol derivative (Sarker et al., 2013) and affected by surface charge, lipophilicity, hydrophobicity, as well as the size (Sarker et al., 2014), have been investigated to deliver bioactive peptides into the cytosolic space of live cells (Sarker et al., 2020). Commercially available cationic liposomes are usually used for the delivery of siRNA (Sarker and Takeoka, 2018). Studies on the structure–activity relationship of cationic lipids have been performed by many research groups (Sarker et al., 2012). However, in order to control the flaws of conventional drug delivery systems, the current research area is expanding its work in the development of modified dosage systems. Hydrogels are under consideration among other novel drug delivery systems. These polymeric networks reduce the limitations of conventional dosage forms and also support a more stable, appropriate, and biocompatible drug delivery system (Karna et al., 2015; Khan et al., 2016). Hydrogels are three-dimensional polymeric networks (Chopra et al., 2022b) that have the capability to absorb water without being dissolved (Chopra et al., 2022a). Hydrogels are comprised of interlinked chains that impart specific mechanical strength. Crosslinking in hydrogels can be achieved physically or chemically, which prevents these grafted networks from dissolving despite absorbing larger quantities of water or biological fluids. It also helps in the release of active moiety at a pre-defined rate by holding them together (Peppas et al., 2000). Bonds in chemically crosslinked hydrogels are generated slowly but are stronger (Sharma et al., 2022). Hydrogels help in the regulation of site-specific drug delivery, which enhances patient compliance by reducing the need for frequent dosing (Ranjha et al., 2010; Singh et al., 2010). The most common applications of hydrogels include their use in tissue and regenerative medicines, separation of biomolecules, biosensors, protein adsorption, agrochemicals, and drug delivery systems (Murali Mohan et al., 2006). Hydrogels in wound dressing have the advantage of possessing the features of moist wound healing with good fluid absorbance (Shawan et al., 2019). The major domain of research in hydrogels is hydrogel-based drug delivery systems, which include sustained-release drug delivery systems, dental applications, injectable polymers, implants, stimuli-responsive systems, and topical applications (Mudassir and Ranjha, 2008). Polymers of natural as well as synthetic origin are being utilized in the pharmaceutical and food industries (Singh et al., 2011). In most cases, one polymer alone is usually unable to regulate the drug release. In order to overcome this drawback, the development of interpenetrating networks presents a better approach for a controlled drug delivery system (Jana et al., 2016). The aloe vera plant, a member of the Liliaceae family, has been found to confer beneficial effects on skin burns, wounds, diabetes, and obesity (Rahman et al., 2017; Minjares-Fuentes et al., 2018). A variety of compounds, including amino acids, carbohydrates, long-chain polysaccharides, vitamin E, vitamin A, and ascorbic acid, are found within aloe vera gel. These bioactive components have effective antioxidant, anti-inflammatory, and antibacterial effects (Salehi et al., 2021). It has a significant action in enhancing the swelling of hydrogels (Guancha-Chalapud et al., 2022) and absorption of the drugs (Haasbroek et al., 2019). *Tamarindus indica* is mostly found in the South East Asian region, especially in India, Pakistan, and Bangladesh. Tamarind seed polysaccharide (TSP) is a galactoxyloglucan in composition (Burgalassi et al., 1996). Its unique properties, such as tolerance to changes in pH, mucoadhesive ability, thermal stability, and biocompatibility, make it a fascinating polysaccharide to be incorporated into drug delivery systems. It is capable of swelling in hot water and forms a thick viscous solution. Tamarind gum has already been evaluated for hydrogel applications, controlled-release tablets, mucoadhesive microsphere synthesis, and nasal, buccal, and ocular applications (Joseph et al., 2012; Prajapati et al., 2013). Tramadol (TRD) is an opioid drug studied for its potent analgesic activity and is being used for pain treatment (Bravo et al., 2017; Subedi et al., 2019). The drug was approved in 1995 by the Food and Drug Administration (FDA) for relief and management of moderate-to-severe pain (Bloor et al., 2012; Vazzana et al., 2015). Tramadol hydrochloride is preferable over classical opioid drugs because of its distinguished pharmacological reputation for exhibiting lower drug abuse potential and lower incidence of side effects (Andurkar et al., 2012; Modi et al., 2013). Due to fast metabolism in the human body, the biological activity of drugs can be relatively short. Therefore, repeated administrations are required in order to maintain the effective drug plasma concentration. The present work was designed to incorporate TRD in biodegradable pH-sensitive AvT-co-poly (MAA) networks synthesized by the free-radical polymerization method. Varying proportions of polymers, monomers, and crosslinkers were used for the synthesis of hydrogels. The study aimed to achieve oral controlled delivery of TRD that will reduce its dosing frequency and enhance patient compliance.

## 2 Materials and methods

### 2.1 Materials

All chemicals used in the research work were of analytical grade. TRD was purchased from Searle (Pvt.) Ltd., Karachi, Pakistan. Aloe vera gel was extracted from plant leaves. Tamarind seed powder was purchased from BDH, England. Methacrylic acid, potassium dihydrogen phosphate, potassium chloride, and ethanol were purchased from Merck, Germany. Ammonium persulfate, methylene-bis-acrylamide, hydrochloric acid, and sodium hydroxide were purchased from Sigma-Aldrich, United States.

### 2.2 Methods

#### 2.2.1 Extraction of aloe vera gel

Fresh leaves of *Aloe barbadensis* were taken from the plant and peeled off, and the gum latex obtained was washed with sufficient quantity of *n*-hexane for 30 min in order to remove fatty oils from the gel. It was then followed by draining the supernatant *n*-hexane. The procedure was repeated thrice. Washed aloe vera gel chunks were shifted to Petri dishes and dried at 45°C in a hot-air oven. After drying, the gel was scrapped off from the petri dishes, ground in pestle and mortar, and stored in well-closed plastic containers for further use (Haseeb et al., 2016).

#### 2.2.2 Synthesis of AvT-co-poly (MAA) hydrogels

An AvT-co-poly (MAA) network was synthesized by using varying concentrations of aloe vera, tamarind, methacrylic acid, and crosslinker, optimizing the free-radical polymerization technique. Both the dried aloe vera gel and tamarind seed powder were separately weighed and soaked in 5–6 ml distilled water for 2 h. Centrifugation of tamarind seed powder (TSP) was carried out at 4000 rpm for 10 min. The supernatant was collected and mixed thoroughly with aloe vera gel dispersion using a magnetic stirrer (VELP Scientifica, Milano, Italy) for 10 min. Methacrylic acid was added drop by drop to the abovementioned mixture with continuous stirring. Solutions of ammonium persulfate (APS) and methylene-bis-acrylamide (MBA) were prepared separately in 5 ml of distilled water. Both solutions were sonicated and incorporated into the abovementioned mixture. During mixing, it was ensured that the mixing of MBA should be accomplished after APS, and the temperature of the reaction mixture was kept at 50°C for 1 h. Nitrogen purging was carried out to remove air bubbles. Afterward, the reaction mixture was transferred into test tubes which were covered with aluminum foil and were kept in a water bath at 65°C for 24 h. Test tubes were then removed from the water bath, and formed hydrogels were recovered by breaking the test tubes. The cylindrical shape was cut into disc shapes with the sharp blade and washed with ethanol and water solution (70:30) to remove unreacted monomers and free radicals. Discs were placed in an oven for drying at 40°C–45°C, which were then stored in well-closed containers, and further studies were executed (Olad et al., 2018) Table 1.

**TABLE 1:** Composition of different formulations of AvT-co-poly (MAA) hydrogels.

Formulation code	Aloe vera (g)	Tamarind gum (g)	Methacrylic acid (g)	Methylene-bis-acrylamide (g)	Ammonium persulfate (g)
AvT1	0.2	0.15	4	0.4	0.3
AvT2	0.4	0.15	4	0.4	0.3
AvT3	0.6	0.15	4	0.4	0.3
AvT4	0.15	0.2	4	0.4	0.3
AvT5	0.15	0.4	4	0.4	0.3
AvT6	0.15	0.6	4	0.4	0.3
AvT7	0.5	0.15	**5**	0.4	0.3
AvT8	0.5	0.15	**6**	0.4	0.3
AvT9	0.5	0.15	**7**	0.4	0.3
AvT10	0.5	0.15	4	**0.5**	0.3
AvT11	0.5	0.15	4	**0.6**	0.3
AvT12	0.5	0.15	4	**0.7**	0.3

*The bold values show the concentrations of ingredients varied during the formulation of hydrogels.

### 2.3 Characterization

#### 2.3.1 Drug loading

TRD was incorporated into hydrogel discs by the swelling-diffusion method. A 1% drug solution in phosphate buffer (pH 7.4) was prepared, and pre-weighed dried hydrogel discs were dipped in the drug solution for 48 h or until they attained constant weight. Discs were then taken out from the solution followed by their washing with distilled water to wash away any drug from the surface of the hydrogels. Drug-loaded discs were dried by keeping them in an oven at 40°C until completely dried and weighed after drying (Shabir et al., 2017).

Percent drug loading was calculated by Eq. 2:

Amount of drug loaded=WD – Wd, (1)

$$Percent\;drug\;loading=\left[\left(WD-Wd\right)/\;Wd\right]\times 100.$$ (2)

Here, W_D_ is the dry weight of the hydrogel disc after drug loading, and W_d_ is the dry weight of the hydrogel disc before loading.

#### 2.3.2 Sol–gel fraction

Sol–gel fraction was determined to find out sol and gel contents of the prepared hydrogel. Sol fraction indicates unreacted contents during the polymerization reaction, whereas gel fraction represents the reactant contents of the crosslinked network. Dried hydrogel discs were weighed and placed in distilled water for 72 h. Interval shaking was carried out to isolate water-soluble contents from the hydrogel. After 72 h, the water-insoluble part of the hydrogels was dried in an oven and carefully weighed (Pandey, Mohd Amin et al., 2013).

The gel and sol contents were then measured as follows:

$$Gel\;fraction\;\left(\%\right)=\left(\frac{W1}{W0}\right)X\;100,$$ (3)

$$Sol\;fraction\;\left(\%\right)=100-Gel\;fraction,$$ (4)

where ‘ W0 ’ is the weight of the dried disc before wetting and ‘W1’is the weight of the dried disc after 72 h of wetting in deionized water.

#### 2.3.3 Determination of swelling ratio

Dried copolymer discs of each formulation were weighed and then soaked in buffer solutions of pH 1.2 and 7.4 (100 ml) at room temperature. Copolymer discs were taken out at regular time intervals, blotted with filter paper, weighed, and soaked again in the respective media (Ranjha et al., 2015). Dynamic swelling (q) was calculated as

q=Ws/Wd, (5)

where ‘ q ' is the dynamic swelling ratio, “Ws” is the weight of swollen gel at time t, and “Wd” represents the initial weight of the sample.

Percent equilibrium swelling (ES) was measured by the following equation:

$$ES\;\left(\%\right)=\left(Meq\;–\;Mo\right)/Meq\times 100,$$ (6)

where ‘ Meq ’ is the mass of the swollen hydrogel at equilibrium and ‘Mo’ is the mass of the dried sample.

#### 2.3.4 On–off responsiveness of the AvT copolymer

The stimuli sensitivity of the copolymer was assessed by the swelling and de-swelling behavior of the AvT hydrogel using the gravimetric method. For this purpose, swelling of the hydrogel was noted in a basic buffer (pH 7.4) for 1 h and then de-swelling in an acidic buffer (pH 1.2) alternately. The study was conducted for 6 consecutive hours (Ashraf et al., 2017).

#### 2.3.5 Scanning electron microscopy analysis

The AvT-co-poly (MAA) network was investigated for its surface morphology by conducting scanning electron microscopy. Analysis was performed by using a scanning electron microscope (JEOL JSM-5910, Japan) at different magnifications (×250-×10000). The hydrogel disc was crushed into fine size and then mounted on an aluminum stub, and a thin layer of gold was coated with a gold sputter coater. Scanning was performed under a high-energy electron beam, and photomicrographs were taken which were further observed for surface morphology evaluation (Hebeish et al., 2013).

#### 2.3.6 Powder X-ray diffraction analysis

The amorphous or crystalline nature of pure TRD, aloe vera, tamarind gum, and the TRD-loaded copolymer was elucidated by performing powder X-ray diffraction analysis. For this, an X-ray analytical X’Pert powder diffractometer was used. Samples were exposed to Cu-Kα radiation, and diffractograms were recorded at an angle 2θ between 0° and 80° by setting the scan rate at 1° per minute (Farhadnejad et al., 2018).

#### 2.3.7 Thermal analysis

Differential scanning calorimetric analysis was performed to determine the thermal degradation pattern of TRD, polymers alone, and the drug-loaded hydrogel disc. The analysis was carried out by operating a thermal analyzer (SDT Q600 TA United States) at a heating range of 0°C–800°C on an aluminum stub under a nitrogen bath (Wang et al., 2019).

#### 2.3.8 Fourier transform infrared spectroscopy

The compatibility of components and formation of the crosslinked polymeric network were determined by performing FTIR spectroscopy (Agilent Cary 630) of pure individual components and the drug-loaded network. It was accomplished by mixing the analyte with KBr powder, and transmittance (cm^-1^) was recorded over a scanning range of (4000–500 cm^−1^) (Larrañeta et al., 2018).

#### 2.3.9 *In vitro* release study of the drug

A drug release study was performed in USP dissolution apparatus-II, using the acidic and basic buffers (pH 1.2 and pH 7.4). TRD-loaded hydrogel discs were placed in each bucket containing 900 ml of buffer solution, and the apparatus was run at 50 rpm at 37°C ± 0.5°C. Samples of 5 ml were drawn with the replacement of the respective buffer and analyzed using a UV spectrophotometer at the wavelength (λmax) 322 nm (Qu et al., 2018). The percent drug release was calculated by the following equation:

$$Drug\;release\;\left(\%\right)=\left(sampleabsorbance/standardabsorbance\right)\times 100.$$ (7)

#### 2.3.10 Kinetic modeling

The mode of release of TRD from the grafted network was investigated by applying the kinetic models. The best-fit model was confirmed by the value of “R^2^,” while the value of “n” described the release mechanism of TRD. Fickian diffusion was proved by the value of n ≤ 0.45, non-Fickian was defined when 0.45 < n > 0.89, and case-II relaxation or super case-II transport was highlighted by n > 0.8.

##### 2.3.10.1 Zero-order kinetics

Zero-order kinetics was determined by using the following equation:

Qt=Q0+K0t. (8)

Here, K_0_ is the TRD release rate constant, Q_0_ is the initial quantity of TR in formulation, and Q_t_ represents the TRD release within the time “t.”

##### 2.3.10.2 First-order kinetics

For the determination of first-order kinetics, the following equation was used:

logW=logWO–kt / 2.303. (9)

Here, W is the amount of drug present in the network, k is the first-order rate constant, *W*_O_ is the initial amount of drug in the hydrogel, and t is the time.

##### 2.3.10.3 Higuchi model

The Higuchi model was determined with the help of the following equation:

Ft=K2t1/2. (10)

Here, F_t_ is the amount of undissolved drug and K_2_ is the Higuchi constant.

##### 2.3.10.4 Korsmeyer–Peppas model

This can be expressed using the following equation:

Ft / F0=k3 tn. (11)

F_t_/F_0_ is the fraction of TRD release at time “t,” K_3_ is the Korsmeyer–Peppas constant, and “n” represents the release exponent.

#### 2.3.11 Acute toxicity studies

An acute toxicity study was performed according to the Organization for Economic Cooperation and Development (OECD) guidelines on animal models (Khan et al., 2021; Badshah et al., 2023). The study was performed in the Faculty of Pharmacy, the University of Lahore. Ethical approval was given by the institutional research ethics committee under reference number IREC-2021-11. Six healthy rabbits having an average weight of 1499 g were purchased from an animal farm, transported to the university animal house, and acclimated for 7 days with a proper diet. Rabbits were divided into two groups, i.e., the control group (CG) and the test group (TG). AvT-co-poly (MAA) discs in a pulverized form (2 g/Kg dose) were administered to the tested group. Animals in both groups were keenly observed for physical activity, any common sign of illness, salvation, diarrhea, skin irritation, mortality, and body weight for 14 days. On the 14th day, animals were weighed again, and the blood samples were withdrawn from the ear marginal vein. These samples were transferred into EDTA tubes, centrifuged at 5000 rpm for plasma separation, and investigated for hematological examination, lipid and renal profiles, and AST and ALT levels. Rabbits were sacrificed after administration of 1 ml/kg dose of anesthesia [ketamine and xylazine (70:30)] for the removal of vital organs. After washing with tap water, a histopathological examination of the internal vital organs (heart, spleen, liver, kidney, small intestine, and lungs) was carried out to detect the toxic effects of the grafted network (Gong et al., 2009).

## 4 Results and discussion

### 4.1 Physical appearance

The formulated AvT hydrogels appeared milky peach in color with a smooth and elastic texture which turned reddish brown upon drying.

### 4.2 Percent drug loading

**FIGURE 1:**
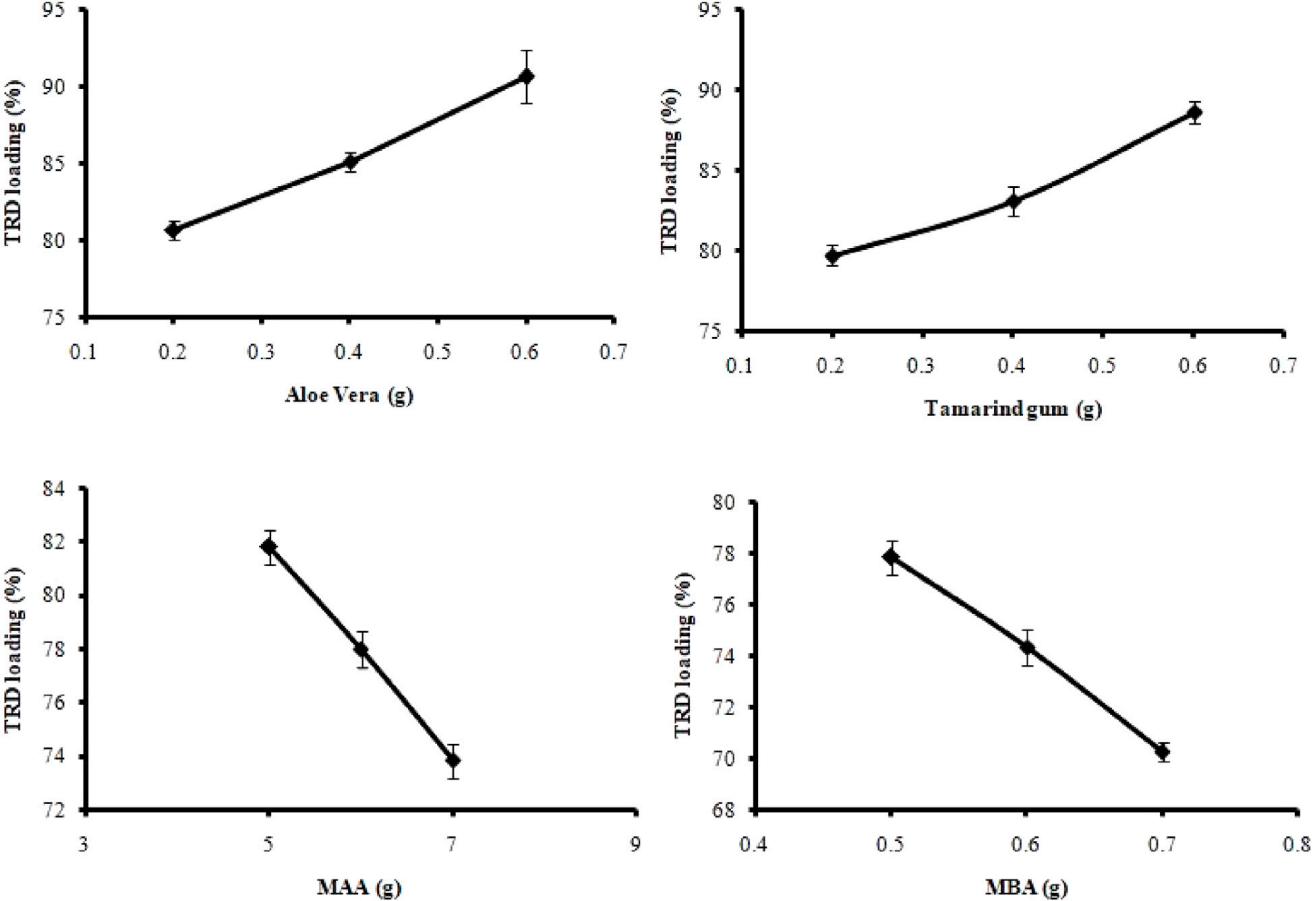
Effect of ingredient concentration on TRD loading.

Loading of TRD in hydrogel discs was performed by swelling and the diffusion-controlled method. All formulations (AvT1–AvT12) resulted in 70.28%–90.64% of drug loading, as presented in Figure 1, and it was affected by varying the amounts of polymers, monomers, and crosslinkers. By consequent increasing of tamarind gum and aloe vera contents in formulations AvT1–AvT3 and AvT4–AvT6, the percent loading of TRD was increased from 80.67% to 90.64%, and 79.72%–88.65%, respectively. Formulations (AvT7–AvT9) with increasing concentrations of MAA showed a decrease in drug loading that ranged from 81.81% to 73.85%. In the case of formulations AvT10–AvT12 having increased contents of MBA, a decrease in drug loading from 77.85% to 70.28% was observed. That decline was due to the formation of a more dense and compact structure by crosslinkers, resulting in low penetration of fluid inside the crosslinked matrix. Bajpai and Giri reported that the KNO_3_ release rate decreased with an increase in the content of MBA (Bajpai and Giri, 2003). AvT3 and AvT6 formulations having maximum concentrations of aloe vera and tamarind gum were found to be optimum, depicting 90.64% and 88.65% of drug loading, while formulations AvT7 and AvT10 containing optimum monomer and crosslinker contents were exhibiting 81.81% and 70.28% of TRD loading, respectively.

### 4.3 Sol–Gel fraction (%)

**FIGURE 2:**
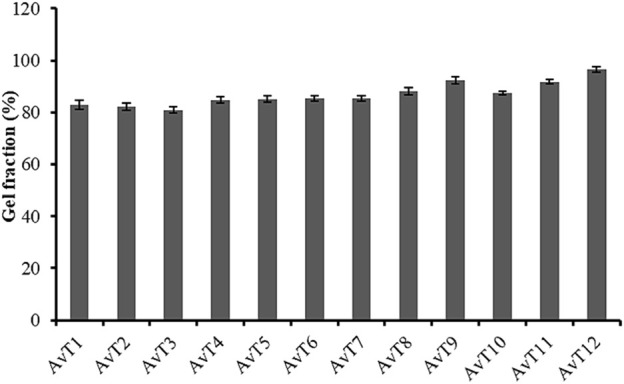
Gel fraction of AvT1–AvT12.

The gel content of the AvT-g-poly (MAA) network was determined in order to analyze the consumption of the reactants during polymerization reactions. The gel fraction of all formulations (AvT1–AvT12) was in the range of 80.95%–96.52%, as shown in Figure 2. Based on the results, the gel fraction of formulations was increased by subsequent increases in the concentrations of the monomer and crosslinker, while no significant effect on gel fraction was observed by increasing the polymer contents. Formulations (AvT1–AvT3) with variable aloe vera contents exhibited a slight decrease in gel fraction of 83.02%–80.95%, while 84.75%–85.37% of gel fraction was shown by formulations (AvT4–AvT6) with increased tamarind gum contents (Shah et al., 2019). However, upon increasing the monomer and crosslinker contents in formulations AvT7–AvT9 and AvT10–AvT12, the percent gel content was found to be increased from 85.51% to 92.39% and from 87.50% to 96.52%, respectively. Crosslinking was promoted by increasing the concentrations of the monomer and crosslinker in the formulations, which resulted in a denser structure with improved network strength. Ranjha et al. (2014) reported a similar increase in the gel fraction of hydrogels on increasing the monomer and crosslinker contents in their study.

### 4.4 Determination of the swelling behavior of the AvT-g-poly (MAA) hydrogel (water absorbency)

The swelling response was studied to investigate the pH responsiveness of hydrogels. Dynamic and percent equilibrium swelling were determined in acidic (pH 1.2) and basic (pH 7.4) buffers. Enhanced swelling was noticed at pH 7.4, while at pH 1.2, all formulations showed minimal swelling. Variations of polymers, monomers, and crosslinker ratios in formulations (AvT1–AvT12) also affected the dynamic and equilibrium swelling of hydrogels.

#### 4.4.1 Effect of varying concentrations of polymers on dynamic swelling

**FIGURE 3:**
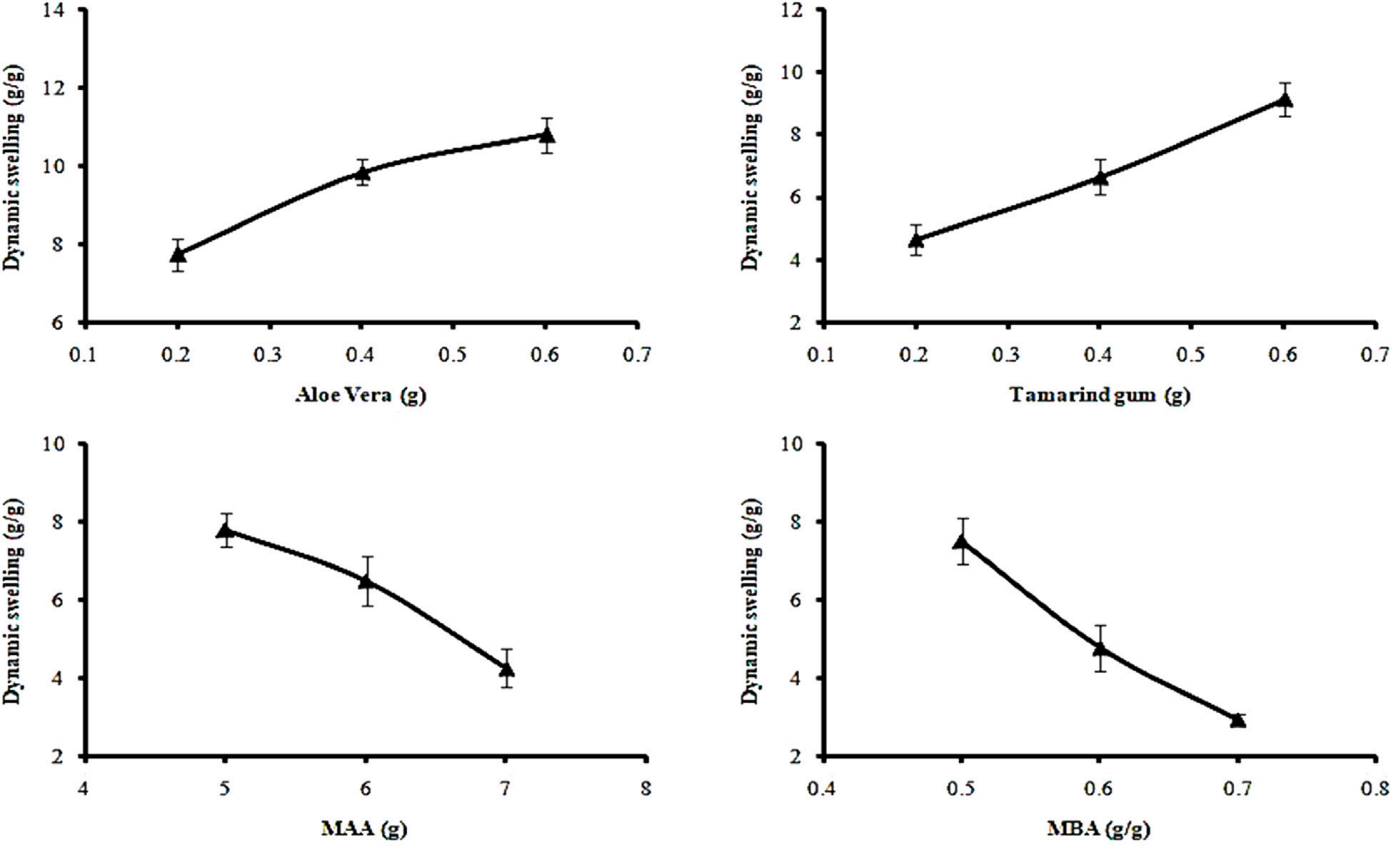
Effect of ingredient concentration on dynamic swelling.

At pH 7.4, dynamic swelling values for AvT1, AvT2, and AvT3 were 7.75, 9.86, and 10.81 g/g, while at pH 1.2, the obtained values were 1.2, 1.89, and 2.43 g/g, respectively, after 24 h (Figure 3). From formulations AvT4–AvT6, swelling ratios were 4.67, 6.67, and 9.14 g/g at pH 7.4, while 0.86, 1.16, and 1.5 g/g were observed at pH 1.2. An increase in both polymer contents in formulations resulted in an enhanced pore volume due to OH groups imparted by the polymers, ultimately facilitating the penetration of water inside the network, contributing to the enhanced swelling ability of hydrogel (Kausar et al., 2021).

#### 4.4.2 Effect of varying concentrations of monomers on dynamic swelling

Formulations (AvT7–AvT9) with increasing concentrations of methacrylic acid concentrations (5–7 g) depicted a decrease in swelling response. The dynamic swelling of formulations ranging from AvT7 to AvT9 at pH 7.4 was 7.80, 6.50, and 4.26 g/g, and at pH 1.2, it was 0.93, 0.81, and 0.68 g/g, respectively, after 24 h (Figure 3). The increase in monomer concentration might be associated with the promotion of inter- and intra-molecular self-crosslinkages, resulting in the formation of a low-porous structure, thus hindering the movement of the solvent into the polymeric matrix (Jindal et al., 2011).

#### 4.4.3 Effect of varying concentrations of crosslinker on dynamic swelling

By increasing the concentration of MBA from 0.5 to 0.7 mg in formulations ranging from AvT10 to Avt12, dynamic swelling was decreased and found to be 8.18, 4.16, and 2.93 g/g at pH 7.4, and 0.82, 0.79, and 0.59 g/g at pH 1.2 after 24 h. Decreased swelling with increased crosslinker content was attributed to improvement in the crosslinking density of the interpenetrating networks, resulting in the development of a more compact and dense structure (Sadeghi and Heidari, 2011; Badshah et al., 2023).

### 4.5 On and off switching of the AvT hydrogel

**FIGURE 4:**
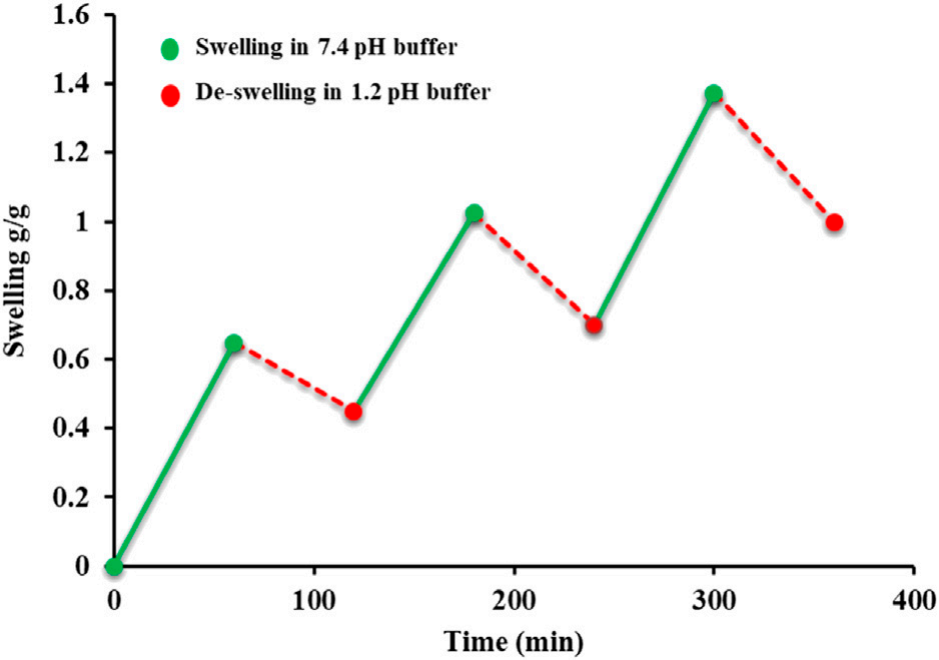
On–off switching of hydrogels.

Swelling and de-swelling experiments were performed at pH 7.4 and pH 1.2, respectively, in a repetitive cycle. All formulation discs showed significant swelling when placed in a basic buffer (pH 7.4), and de-swelling was exhibited by these swollen discs when immersed in an acidic buffer (1.2), as represented in Figure 4. These swelling and de-swelling behaviors were due to the stimuli-responsive nature of the AvT-g-poly (MAA) network system. At basic pH, deprotonation of COOH⸻ groups imparted by MAA in hydrogel chains resulted in repulsion within functional units, thus facilitating the pore opening and swelling response, whereas at acidic pH, these functional groups became protonated, causing the de-swelling of hydrogels due to reduced repulsive forces of polymeric chains (Ashraf et al., 2017).

### 4.6 Scanning electron microscope analysis

**FIGURE 5:**
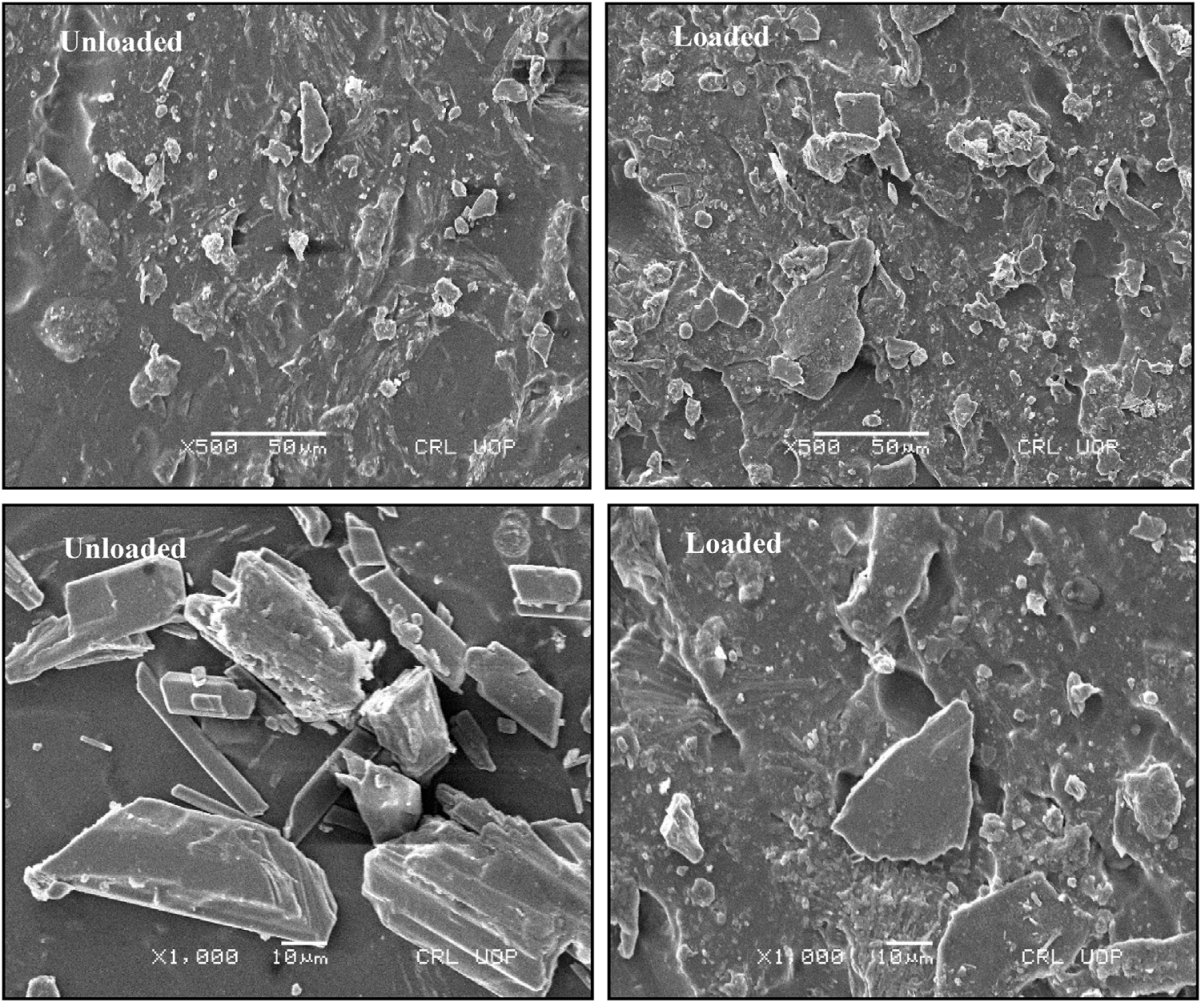
SEM photomicrographs of TRD-loaded and unloaded hydrogels.

The formulated pH-responsive hydrogels were evaluated for surface morphology through scanning electron microscopy. Photomicrographs obtained at various resolutions showed rough outer surfaces that might be due to cutting of hydrogel discs and also showed adherence to the drug over the surface. Moreover, the surface images account for the presence of pores and cracks within polymeric structures, as shown in Figure 5. The porous structure is significantly feasible for maximum solubility and dissolution of drugs (Badshah et al., 2021). The presence of pores facilitates the movement of water and other biological fluids into and out of the hydrogel network. By increasing crosslinking density, pores were decreased in the network mesh and *vice versa*. Porous structures accompanied by ionic and hydrophilic groups of hydrogel constituents are responsible for the elaborative swelling of IPN. Cracks may be formed during drying due to partial collapse of the polymeric gel network. Similar morphology having rough surfaces with pores and cracks on tamarind gum-based mucoadhesive beads was also found in a previous study (Nayak et al., 2014).

### 4.7 Powder X-ray diffraction

**FIGURE 6:**
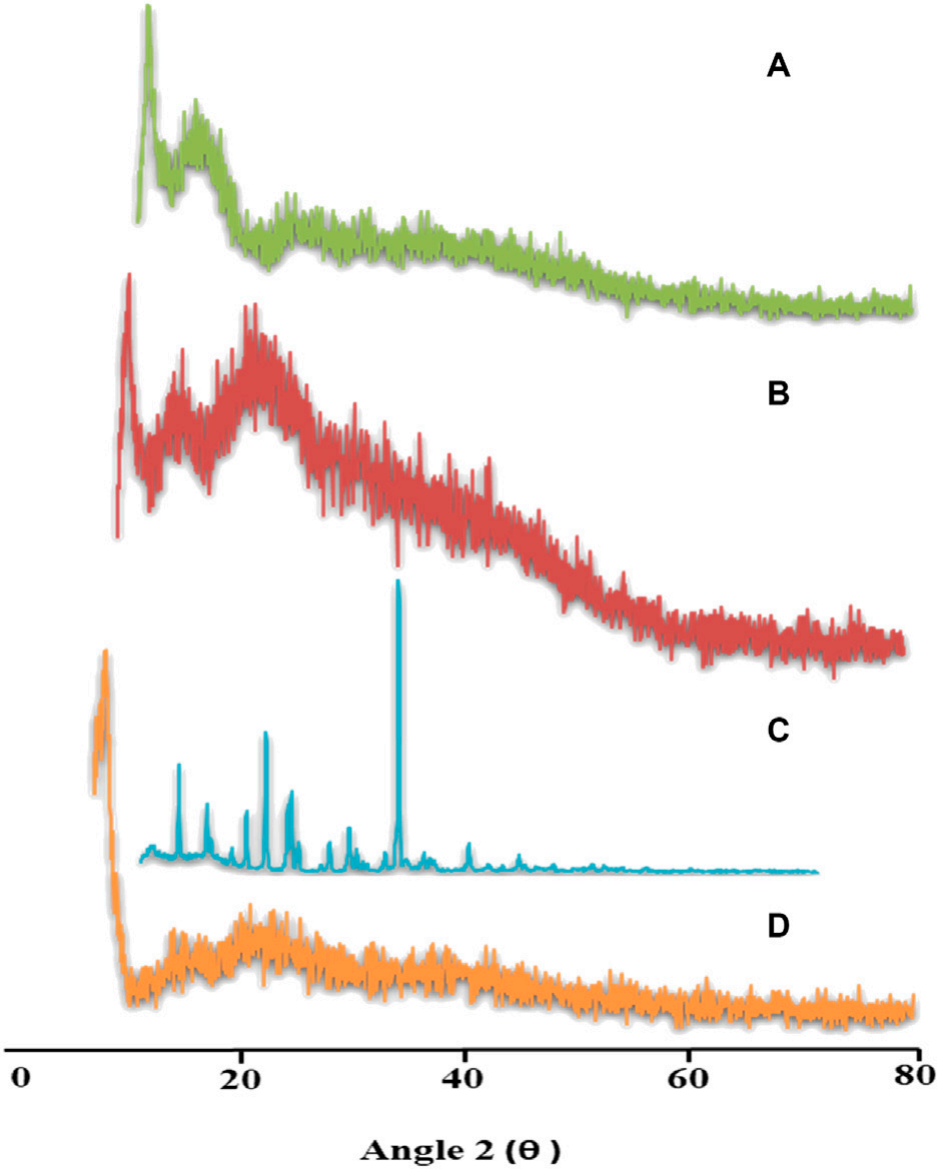
XRD patterns of aloe vera **(A)**, tamarind gum **(B)**, TR **(C),** and TR-loaded hydrogels **(D)**.

Distinct characteristic peaks of TRD were presented at 2θ = 10.6°, 18.6°, 20.95°, and 30.95° on a powder X-Ray diffractogram, which clearly indicated the crystalline nature of the drug. Diffractograms of aloe vera and tamarind gum displayed fused curves having no sharp peaks, thus describing the amorphous nature of both polymers. In the case of TRD-loaded hydrogels, all intense peaks of the drug were converted into fused curves demonstrating the amorphous dispersion of the drug within the network (Figure 6). These results revealed the improved solubility of the drug when incorporated into polymeric networks, as indicated by the merged peaks of the drug displayed on drug-loaded hydrogels (Sarfraz et al., 2018).

### 4.8 Differential scanning calorimetry

**FIGURE 7:**
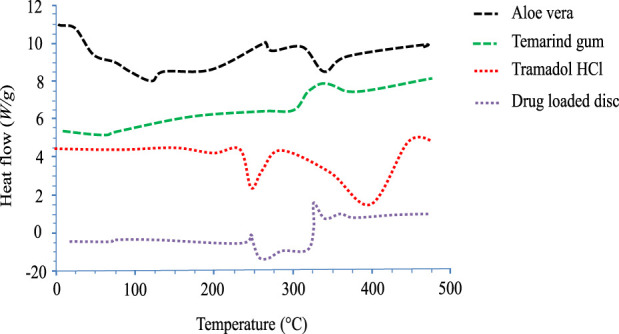
DSC thermograms of aloe vera, tamarind gum, tramadol HCl, and the drug-loaded hydrogel disc.

DSC analysis of pure drugs, polymers, and fabricated networks was conducted to investigate their thermal nature (Figure 7). The thermogram of pure TRD displayed an endothermic peak between 180–185°C, indicating the melting point of the drug (Jeevana and Sunitha, 2009). In the case of aloe vera, a slight endothermic peak appeared at 100°C, which was due to the loss of moisture content from the polymer. Another broad endothermic peak at 300°C–400°C was attributed to the thermal degradation of cellulose and lignin in aloe vera (Aghamohamadi et al., 2019; Andonegi et al., 2020). The DSC thermogram for tamarind gum has revealed an endothermic peak from about 30°C to 70°C, showing moisture loss from the polymer. Other peaks displayed at 305°C and 450°C on the thermogram were depicting the melting range and thermal decomposition of the polymer, respectively. TRD-loaded hydrogels were also investigated for thermal analysis, showing an endothermic peak at 200°C–205°C, an exothermic peak at 250°C, and transformation of some major peaks of drug and polymers into minor ones. Shifting of peaks on the DSC thermogram of drug-loaded hydrogel confirmed the amorphous form as well as the thermal stability of the polymeric blend.

### 4.9 Fourier transform infrared spectroscopy

**FIGURE 8:**
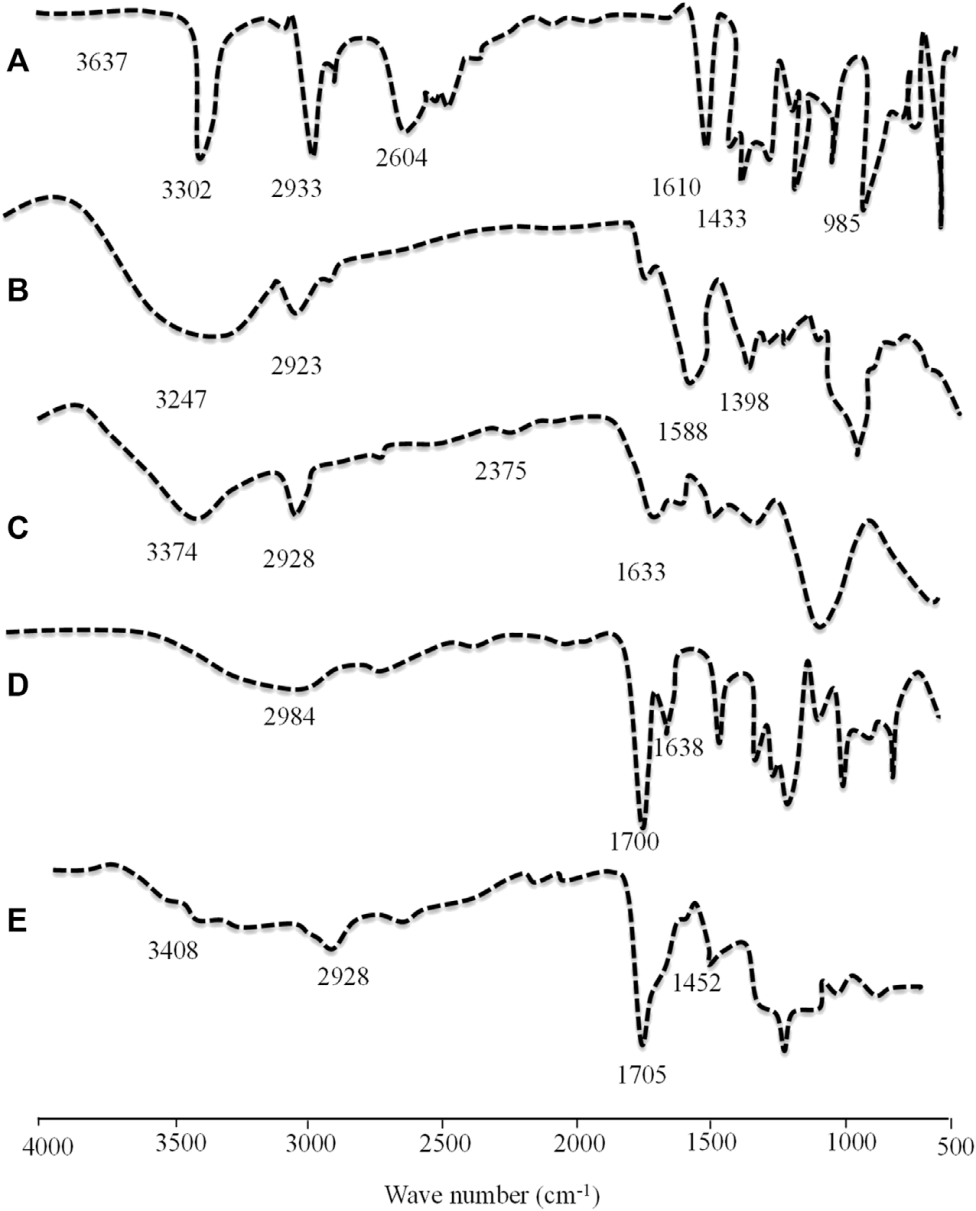
FTIR spectra of TRD **(A)**, aloe vera **(B)**, tamarind gum **(C)**, methacrylic acid **(D)**, and the TRD-loaded hydrogel **(E)**.

Structural and compositional features of the grafted network were confirmed by performing Fourier transform infrared (FTIR) spectroscopy of drugs, polymers, monomers, and TRD-loaded hydrogels. The FTIR spectra of all components are displayed in Figure 8. The FTIR spectrum of pure TRD showed characteristic evident peaks at 1433.46 cm^−1^, 1610.04 cm^−1^, 2933.03 cm^−1^, and 3302.03 cm^−1^ describing CH_3_ stretching, conjugated C=O, CH asymmetric stretching, and Ar-NH_2_, respectively (Choudhury, 2020). For aloe vera gel, some prominent peaks were observed at 1024 cm^−1^, 1398.02 cm^−1^, 1588 cm^−1^, and 2922.06 cm^−1^ on the FTIR spectrum that were corresponding to C–O stretching for ether, CN stretching, NH bending, and CH stretching of aliphatic alkanes, respectively. Moreover, another absorption band at 3246.40 cm^−1^ was associated with NH-stretching vibration (Swami Hulle et al., 2014; Dey et al., 2015). The FTIR spectrum of tamarind gum has displayed intense peaks at 1035 cm^−1^ (CH-OH) stretching), 1633 cm^−1^ (carbonyl group (-HC = O) stretching), 2402 cm^−1^, and 2928.05 cm^−1^ (intramolecular and intermolecular hydrogen bonding). Another extended peak at 3274.05 cm^−1^ was confirming the presence of a -OH group within the polymeric chains of tamarind gum (Abd El-Mohdy et al., 2016). Characteristic bands of MAA were presented at 1638 cm^−1^, 1700 cm^−1^, and 2984 cm^−1^ on the FTIR spectrum that were associated with C=C stretching vibrations, carboxylic acid groups, and methyl C–H asymmetric stretching, respectively. In the case of the FTIR spectrum of the TRD-loaded hydrogel, prominent peaks of drug and polymers were observed with slight variations in intensities and positions. Evident peaks of TRD at 1433 cm^−1^ and 2933 cm^−1^ due to CH_3_ stretching and CH asymmetric stretching were displaced to 1452 cm^−1^ and 2928 cm^−1^ on the spectrum of the drug-loaded hydrogel, confirming the encapsulation of the drug within the matrix. Similarly, the tamarind gum peak at 3274.05 cm^−1^ was shifted towards a higher wave number, i.e., 3500 cm^−1^, on the grafted network. These displacements and variations in the intensities of certain peaks on the spectrum of drug-loaded hydrogels were proving the complexation and compatibility of developed network components.

### 4.10 *In vitro* release studies

**FIGURE 9:**
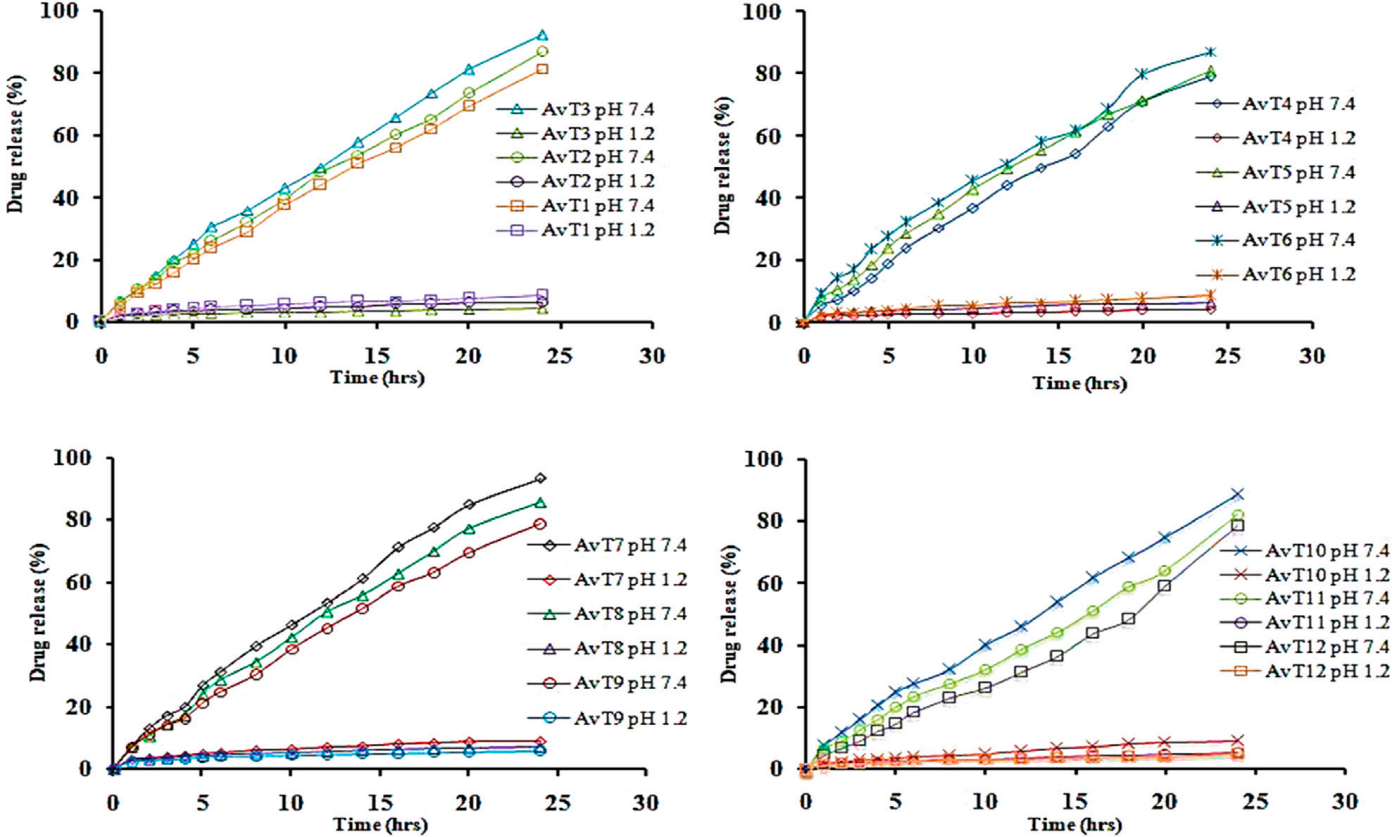
TRD release from formulations at pH 1.2 and pH 7.4.

*In vitro* release of TRD from the grafted network was performed at acidic and basic pH (1.2 and 7.4), and the effects of aloe vera, tamarind gum, MAA, and MBA concentrations on the release of drug were determined (Figure 9 **)**. A gradual increase in the release of TRD was observed by sequentially increasing the aloe vera and tamarind gum concentrations in formulations from AvT1–AvT3 and AvT4–AvT6, ranging within 81.46%–92.22% and 79.13%–87.27%, respectively. The pore volume of the network was increased by increasing the polymer contents, causing penetration of the excessive-dissolution fluid inside the network, resulting in higher swelling and release of TRD from the grafted system (Kausar et al., 2021). With an increase in MAA concentration in formulations from AvT7 to AvT9, a decrease in the release of the drug from 91.81%–83.02% was noticed. The decline in drug release was attributed to stronger inter- and intramolecular forces within the matrix by the monomer, thus reducing the porous network and limiting the passage of fluid inside the hydrogel network. Likewise, the release of TRD was also decreased, ranging within 89.01%–78.43% for formulations AvT10–AvT12 having an increased concentration of crosslinker content. Optimum results were obtained by formulations AvT3, AvT6, AvT7, and AvT10, showing 92.22%, 87.27%, 91.81%, and 78.43% of drug release at pH 7.4.

### 4.11 Kinetic modeling

Release kinetics was further studied by the application of different kinetic models, i.e., the zero-order, first-order, Higuchi, and Korsmeyer–Peppas model, through the DD-solver built-in application. For all formulations (AvT1–AvT12), zero order was noted to be the best-fit model, as defined by the R^2^ value (Table 2). The mode of TRD release was revealed from the value of “n,” which was super case-II transport for most of the formulations.

**TABLE 2:** Kinetic modeling on TRD release data.

Formulation	Zero order	1st order	Higuchi	Korsmeyer–Peppas	
	R^2^	R^2^	R^2^	R^2^	n
AvT1	0.9969	0.9576	0.8798	0.9994	0.900
AvT2	0.9973	0.9745	0.8911	0.9989	0.870
AvT3	0.9881	0.9682	0.8947	0.9987	0.859
AvT4	0.9930	0.9721	0.8703	0.9973	0.917
AvT5	1.000	0.9586	0.9194	0.9977	0.788
AvT6	0.9933	0.9400	0.9351	0.9971	0.748
AvT7	0.9758	0.9656	0.9067	0.9977	0.823
AvT8	0.9809	0.9762	0.9001	0.9980	0.841
AvT9	0.9982	0.9536	0.9013	0.9983	0.840
AvT10	0.9843	0.9697	0.8995	0.9969	0.846
AvT11	0.9898	0.9603	0.8651	0.9918	0.933
AvT12	0.9790	0.9283	0.7977	0.9840	1.124

### 4.12 Acute oral toxicity

**FIGURE 10:**
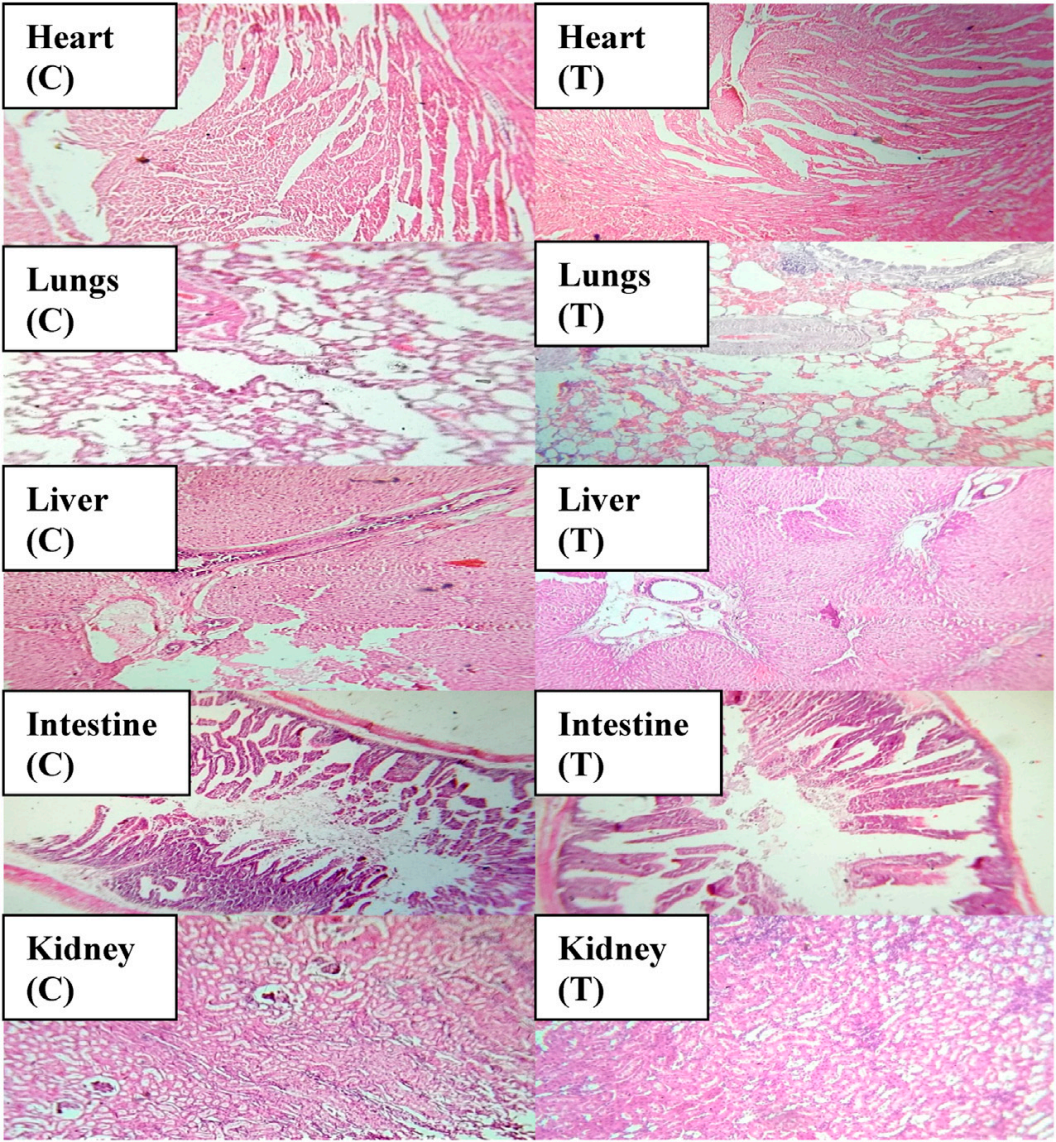
Histopathological examination of vital organs of control and tested groups.

Toxicological evaluation of the AvT-co-poly (MAA) network was carried out by conducting toxicity studies in healthy rabbits. During the toxicity study, both groups (control and tested) of animals were closely observed for body weight, water and food intake, and clinical findings, i.e., heart rate, skin rashes, fever, and behavioral changes, for 14 days. There was no indication of ocular and dermal toxicity or remarkable weight variation. Similarly, zero mortality rate was found in both groups, as reported in Table 3. The biocompatibility of the grafted system was proved by the results of hematological and biochemical analyses of blood samples, showing all values within acceptable ranges, as documented in Tables 4, 5. The newly grafted system was investigated for its toxicity by carrying out histopathological examinations of vital organs, i.e., the liver, heart, lung, kidney, and intestine. After sacrificing the rabbits on the 14th day, these organs were removed, and tissue slides were prepared and examined microscopically. As observed from the photomicrographs presented in Figure 10, there was no sign of swelling, lesions, abrasion, or deformation found in the vital organs of both tested and control groups. Histopathological examination of the liver revealed a precise arrangement of the hepatic cord and lobules without showing any disruption or abnormality. Heart tissue displayed normal myocytes with no signs of inflammation or necrosis in both groups. The lungs of both treated and control groups were observed to be normal without having any degeneration or lesions. Moreover, kidney tissues were visible with proper bowman capsule, glomerulus, and tubules, showing no hemorrhage and disruption of cells. Similarly, a healthy intestine was seen with entire muscularis and columnar epithelium in both groups, as shown by the microscopic examination. There was no significant difference observed in the organs of both groups, hence proving the biocompatibility and safety of the AvT-co-poly (MAA) network (Mahmood et al., 2016).

**TABLE 3:** Clinical findings of animals during toxicity studies.

Observations	Controlled group	Tested group
Signs of illness	Not observed	Not observed
Body weight (Kg)		
Pre-treatment	1472.67 ± 107.15	1456 ± 117.63
Day 1	1466.18 ± 100.87	1456.03 ± 91.87
Day 7	1469.91 ± 103.15	1469.84 ± 112.43
Day 14	1470.15 ± 98.54	1471.05 ± 99.16
Water intake (ml)		
Pre-treatment	203.1 ± 2.45	204.59 ± 3.38
Day 1	197.54 ± 0.95	201.35 ± 1.07
Day 7	196.08 ± 1.76	198.06 ± 1.34
Day 14	196 ± 1.15	197.16 ± 2.15
Food intake (g)		
Pre-treatment	74.16 ± 1.35	77.11 ± 1.38
Day 1	76.59 ± 1.36	78.01 ± 1.63
Day 7	77.03 ± 1.42	79.48 ± 1.46
Day 14	79.04 ± 1.68	80.88 ± 1.61
Dermal toxicity	Absent	Absent
Ocular toxicity	Not found	Not found
Mortality	Nil	Nil

**TABLE 4:** Results of the hematological analysis of rabbit blood.

Parameters	Controlled group (control)	Treated group (treated)
Hemoglobin (g/dl)	11.12	11.60
pH	7.25 ± 0.14	7.08 ± 0.26
Red blood cells (×10^6^/µl)	5.92±1.45	5.79±1.22
White blood cells (×10^9^L^−1^)	5.7± 0.72	6.2± 1.34
Monocytes (%)	3.45 ± 1.45	3.72 ± 0.77
Platelets (×10^9^L^−1^)	4.72 ± 0.33	4.99 ± 0.34
Lymphocytes (%)	70.9± 1.62	68.6± 0.81
Neutrophils (%)	50.254 ± 2.12	51.52 ± 0.73
Mean corpuscular hemoglobin (pg/cell)	19.4± 0.53	18.3± 1.12
Mean corpuscular volume (%)	70.2± 1.35	73.4± 1.61
Mean corpuscular hemoglobin conc. (%)	34.6± 2.21	33.9± 1.33

**TABLE 5:** Results of LFTs and RFTs of controlled and treated groups.

Biochemical analysis	Group A (control)	Group B (treated)
ALT (U/L)	59± 0.65	75± 1.71
AST (U/L)	74± 1.37	60 ± 2.21
ALP(U/L)	85± 2.11	93± 0.41
Albumin (g/dl)	4.2± 1.26	4.3± 2.07
Total protein (g/dl)	6± 0.21	6± 0.18
Urea (mmol/L)	14.23 ± 0.47	15.36 ± 1.54
Creatinine (mg/dL)	0.6 ± 0.45	0.5 ± 0.34
Uric acid (mg/dL)	3.72 ± 0.47	3.95 ± 1.25
Cholesterol (mg/dL)	61.13 ± 1.31	58.75 ± 1.71
Triglycerides (mg/dL)	60.62 ± 1.36	55.41 ± 2.25

## 5 Conclusion

Aloe vera and tamarind gum were copolymerized by employing MAA and MBA to develop AvT-co-poly (MAA) hydrogels and loaded with TRD. The grafted system was proved to be thermally stable, as confirmed through DSC analysis. FTIR findings displayed the compatibility and complexation of hydrogel components. The transformation of the crystalline character of TRD into an amorphous one was validated by PXRD studies. Swelling of hydrogels, TRD loading, and release rate were dependent on the amounts of polymers, monomers, and crosslinkers in the formulations. The pH responsiveness of the network was also confirmed as maximum swelling, loading, and release of TRD were found at basic pH. A maximum release of 92.22% was shown over the period of 32 h, thus displaying the controlled drug delivery. Moreover, the biocompatibility of the carrier system was indicated by the results of acute oral toxicity studies in healthy rabbits, displaying no signs of illness.

## Data Availability

The original contributions presented in the study are included in the article/Supplementary Material; further inquiries can be directed to the corresponding authors.

## References

[B1] Abd El-Mohdy H. Hegazy E. El-Nesr E. El-Wahab M. (2016). Synthesis, characterization and properties of radiation-induced Starch/(EG-co-MAA) hydrogels. Arabian J. Chem. 9, S1627–S1635. 10.1016/j.arabjc.2012.04.022

[B2] Aghamohamadi N. Sanjani N. S. Majidi R. F. Nasrollahi S. A. (2019). Preparation and characterization of Aloe vera acetate and electrospinning fibers as promising antibacterial properties materials. Mater. Sci. Eng. C 94, 445–452. 10.1016/j.msec.2018.09.058 30423728

[B3] Andonegi M. Irastorza A. Izeta A. de la Caba K. Guerrero P. (2020). Physicochemical and biological performance of aloe vera-incorporated native collagen films. Pharmaceutics 12 (12), 1173. 10.3390/pharmaceutics12121173 33276436 PMC7760042

[B4] Andurkar S. V. Gendler L. Gulati A. (2012). Tramadol antinociception is potentiated by clonidine through α2-adrenergic and I2-imidazoline but not by endothelin ETA receptors in mice. Eur. J. Pharmacol. 683 (1-3), 109–115. 10.1016/j.ejphar.2012.03.016 22449379

[B5] Ashraf M. U. Hussain M. A. Muhammad G. Haseeb M. T. Bashir S. Hussain S. Z. (2017). A superporous and superabsorbent glucuronoxylan hydrogel from quince (Cydonia oblanga): Stimuli responsive swelling, on-off switching and drug release. Int. J. Biol. Macromol. 95, 138–144. 10.1016/j.ijbiomac.2016.11.057 27865952

[B6] Badshah S. F. Akhtar N. Minhas M. U. Khan K. U. Khan S. Abdullah O. (2021). Porous and highly responsive cross-linked β-cyclodextrin based nanomatrices for improvement in drug dissolution and absorption. Life Sci. 267, 118931. 10.1016/j.lfs.2020.118931 33359243

[B7] Badshah S. F. Minhas M. U. Khan K. U. Barkat K. Abdullah O. Munir A. (2023). Structural and *in-vitro* characterization of highly swellable β-cyclodextrin polymeric nanogels fabricated by free radical polymerization for solubility enhancement of rosuvastatin. Part. Sci. Technol., 1–15. 10.1080/02726351.2023.2183161

[B8] Bajpai A. K. Giri A. (2003). Water sorption behaviour of highly swelling (carboxy methylcellulose-g-polyacrylamide) hydrogels and release of potassium nitrate as agrochemical. Carbohydr. Polym. 53 (3), 271–279. 10.1016/s0144-8617(03)00071-7

[B9] Bloor M. Paech M. Kaye R. (2012). Tramadol in pregnancy and lactation. Int. J. obstetric Anesth. 21 (2), 163–167. 10.1016/j.ijoa.2011.10.008 22317891

[B10] Brahmareddy D. Madhulata A. Priyanka R. (2015). Formulation and evaluation of venlafaxine HCl sustained release pellets. Int. J. Pharm. Sci. 5 (6), 632–635.

[B11] Bravo L. Mico J. A. Berrocoso E. (2017). Discovery and development of tramadol for the treatment of pain. Expert Opin. drug Discov. 12 (12), 1281–1291. 10.1080/17460441.2017.1377697 28920461

[B12] Burgalassi S. Panichi L. Saettone M. F. Jacobsen J. Rassing M. (1996). Development and *in vitro*/*in vivo* testing of mucoadhesive buccal patches releasing benzydamine and lidocaine. Int. J. Pharm. 133 (1-2), 1–7. 10.1016/0378-5173(95)04392-6

[B13] Chopra H. Bibi S. Kumar S. Khan M. S. Kumar P. Singh I. (2022a). Preparation and evaluation of chitosan/PVA based hydrogel films loaded with honey for wound healing application. Gels 8 (2), 111. 10.3390/gels8020111 35200493 PMC8871709

[B14] Chopra H. Singh I. Kumar S. Bhattacharya T. Rahman M. H. Akter R. (2022b). A comprehensive review on hydrogels. Curr. Drug Deliv. 19 (6), 658–675. 10.2174/1567201818666210601155558 34077344

[B15] Choudhury S. (2020). Formulation, development and evaluation of sustained release matrix tablet of tramadol hydrocholoride using various hydrophilic natural polymers. Int. Res. J. Pharm. Med. Sci. 3, 5–10.

[B16] Dey A. Bera R. Chakrabarty D. (2015). Influence of Aloe vera on the properties of N-vinylpyrrolidone-Acrylamide copolymer hydrogel. Mater. Chem. Phys. 168, 168–179. 10.1016/j.matchemphys.2015.11.017

[B17] Farhadnejad H. Mortazavi S. A. Erfan M. Darbasizadeh B. Motasadizadeh H. Fatahi Y. (2018). Facile preparation and characterization of pH sensitive Mt/CMC nanocomposite hydrogel beads for propranolol controlled release. Int. J. Biol. Macromol. 111, 696–705. 10.1016/j.ijbiomac.2018.01.061 29337099

[B18] Gong C. Y. Shi S. Dong P. W. Yang B. Qi X. R. Guo G. (2009). Biodegradable *in situ* gel-forming controlled drug delivery system based on thermosensitive PCL–PEG–PCL hydrogel: Part 1—synthesis, characterization, and acute toxicity evaluation. J. Pharm. Sci. 98 (12), 4684–4694. 10.1002/jps.21780 19367619

[B19] Guancha-Chalapud M. A. Serna-Cock L. Tirado D. F. (2022). Aloe vera rind valorization to improve the swelling capacity of commercial acrylic hydrogels. Fibers 10 (9), 73. 10.3390/fib10090073

[B20] Haasbroek A. Willers C. Glyn M. du Plessis L. Hamman J. (2019). Intestinal drug absorption enhancement by aloe vera gel and whole leaf extract: *In vitro* investigations into the mechanisms of action. Pharmaceutics 11 (1), 36. 10.3390/pharmaceutics11010036 30669246 PMC6359586

[B21] Haseeb M. T. Hussain M. A. Yuk S. H. Bashir S. Nauman M. (2016). Polysaccharides based superabsorbent hydrogel from Linseed: Dynamic swelling, stimuli responsive on–off switching and drug release. Carbohydr. Polym. 136, 750–756. 10.1016/j.carbpol.2015.09.092 26572409

[B22] Hebeish A. Hashem M. Abd El-Hady M. Sharaf S. (2013). Development of CMC hydrogels loaded with silver nano-particles for medical applications. Carbohydr. Polym. 92 (1), 407–413. 10.1016/j.carbpol.2012.08.094 23218313

[B23] Jana S. Sharma R. Maiti S. Sen K. K. (2016). Interpenetrating hydrogels of O-carboxymethyl Tamarind gum and alginate for monitoring delivery of acyclovir. Int. J. Biol. Macromol. 92, 1034–1039. 10.1016/j.ijbiomac.2016.08.017 27514441

[B24] Jeevana J. Sunitha G. (2009). Development and evaluation of gelatin microspheres of tramadol hydrochloride. J. Young Pharm. 1 (1), 24. 10.4103/0975-1483.51871

[B25] Jindal R. Kaith B. S. Mittal H. (2011). Rapid synthesis of acrylamide onto xanthan gum based hydrogels under microwave radiations for enhanced thermal and chemical modifications. Polym. Renew. Resour. 2 (3), 105–116. 10.1177/204124791100200302

[B26] Joseph J. Kanchalochana S. Rajalakshmi G. Hari V. Durai R. D. (2012). Tamarind seed polysaccharide: A promising natural excipient for pharmaceuticals. Int. J. Green Pharm. (IJGP) 6 (4), 270. 10.4103/0973-8258.108205

[B27] Karna S. Chaturvedi S. Agrawal V. Alim M. (2015). Formulation approaches for sustained release dosage forms: A review. Asian J. Pharm. Clin. Res. 8, 34–41.

[B28] Kausar S. Erum A. Tulain U. R. Hussain M. A. Farid-ul-Haq M. Malik N. S. (2021). Formulation, *in vitro* evaluation, and toxicity studies of A. Vulgaris-co-AAm carrier for vildagliptin. Adv. Polym. Technol. 1, 1–17. 10.1155/2021/6634780

[B29] Khan K. U. Minhas M. U. Sohail M. Badshah S. F. Abdullah O. Khan S. (2021). Synthesis of PEG-4000-co-poly (AMPS) nanogels by cross-linking polymerization as highly responsive networks for enhancement in meloxicam solubility. Drug Dev. Industrial Pharm. 47 (3), 465–476. 10.1080/03639045.2021.1892738 33651645

[B30] Khan S. Ullah A. Ullah K. Rehman N.-u. (2016). Insight into hydrogels. Des. monomers Polym. 19 (5), 456–478. 10.1080/15685551.2016.1169380

[B31] Larrañeta E. Henry M. Irwin N. J. Trotter J. Perminova A. A. Donnelly R. F. (2018). Synthesis and characterization of hyaluronic acid hydrogels crosslinked using a solvent-free process for potential biomedical applications. Carbohydr. Polym. 181, 1194–1205. 10.1016/j.carbpol.2017.12.015 29253949 PMC5742632

[B32] Mahmood A. Ahmad M. Sarfraz R. M. Minhas M. U. (2016). β-CD based hydrogel microparticulate system to improve the solubility of acyclovir: Optimization through *in-vitro*, *in-vivo* and toxicological evaluation. J. Drug Deliv. Sci. Technol. 36, 75–88. 10.1016/j.jddst.2016.09.005

[B33] Minjares-Fuentes R. Femenia A. Comas-Serra F. Rodríguez-González V. M. (2018). Compositional and structural features of the main bioactive polysaccharides present in the Aloe vera plant. J. AOAC Int. 101 (6), 1711–1719. 10.5740/jaoacint.18-0119 29895349

[B34] Modi H. Mazumdar B. Bhatt J. (2013). Study of interaction of tramadol with amlodipine in mice. Indian J. Pharmacol. 45 (1), 76. 10.4103/0253-7613.106440 23543914 PMC3608300

[B35] Mudassir J. Ranjha N. M. (2008). Dynamic and equilibrium swelling studies: Crosslinked pH sensitive methyl methacrylate-co-itaconic acid (MMA-co-IA) hydrogels. J. Polym. Res. 15 (3), 195–203. 10.1007/s10965-007-9159-x

[B36] Murali Mohan Y. Sudhakar K. Keshava Murthy P. Mohan Raju K. (2006). Swelling properties of chemically crosslinked poly (acrylamide-co-maleic acid) hydrogels. Int. J. Polym. Mater. 55 (7), 513–536. 10.1080/00914030500208246

[B37] Nayak A. K. Pal D. Santra K. (2014). Development of calcium pectinate-tamarind seed polysaccharide mucoadhesive beads containing metformin HCl. Carbohydr. Polym. 101, 220–230. 10.1016/j.carbpol.2013.09.024 24299768

[B38] Olad A. Zebhi H. Salari D. Mirmohseni A. Reyhanitabar A. (2018). A promising porous polymer-nanoclay hydrogel nanocomposite as water reservoir material: Synthesis and kinetic study. J. Porous Mater. 25 (3), 665–675. 10.1007/s10934-017-0479-x

[B39] Pandey M. Mohd Amin M. C. I. Ahmad N. Abeer M. M. (2013). Rapid synthesis of superabsorbent smart-swelling bacterial cellulose/acrylamide-based hydrogels for drug delivery. Int. J. Polym. Sci. 2013, 1–10. 10.1155/2013/905471

[B40] Peppas N. A. Bures P. Leobandung W. Ichikawa H. (2000). Hydrogels in pharmaceutical formulations. Eur. J. Pharm. Biopharm. 50 (1), 27–46. 10.1016/s0939-6411(00)00090-4 10840191

[B41] Prajapati V. D. Jani G. K. Moradiya N. G. Randeria N. P. (2013). Pharmaceutical applications of various natural gums, mucilages and their modified forms. Carbohydr. Polym. 92 (2), 1685–1699. 10.1016/j.carbpol.2012.11.021 23399207

[B42] Qu J. Zhao X. Ma P. X. Guo B. (2018). Injectable antibacterial conductive hydrogels with dual response to an electric field and pH for localized “smart” drug release. Acta biomater. 72, 55–69. 10.1016/j.actbio.2018.03.018 29555459

[B43] Rahman S. Carter P. Bhattarai N. (2017). Aloe vera for tissue engineering applications. J. Funct. biomaterials 8 (1), 6. 10.3390/jfb8010006 PMC5371879 28216559

[B44] Ranjha N. M. Ayub G. Naseem S. Ansari M. T. (2010). Preparation and characterization of hybrid pH-sensitive hydrogels of chitosan-co-acrylic acid for controlled release of verapamil. J. Mater. Sci. Mater. Med. 21 (10), 2805–2816. 10.1007/s10856-010-4134-1 20686825

[B45] Ranjha N. M. Hanif M. Afzal Z. Abbas G. (2015). Diffusion coefficient, porosity measurement, dynamic and equilibrium swelling studies of acrylic acid/polyvinyl alcohol (aa/pva) hydrogels. Pak. J. Pharm. Sci. 1, 48–57. 10.22200/pjpr.2015248-57

[B46] Ranjha N. M. Madni A. Bakar A. A. Talib N. Ahmad S. Ahmad H. (2014). Preparation and characterization of isosorbide mononitrate hydrogels obtained by free-radical polymerization for site-specific delivery. Trop. J. Pharm. Res. 13 (12), 1979–1985. 10.4314/tjpr.v13i12.4

[B47] Sadeghi M. Heidari B. (2011). Crosslinked graft copolymer of methacrylic acid and gelatin as a novel hydrogel with pH-responsiveness properties. Materials 4 (3), 543–552. 10.3390/ma4030543 28880004 PMC5448497

[B48] Salehi M. Zamiri S. Samadian H. Ai J. Foroutani L. Ai A. (2021). Chitosan hydrogel loaded with *Aloe vera* gel and tetrasodium ethylenediaminetetraacetic acid (EDTA) as the wound healing material: *In vitro* and *in vivo* study. J. Appl. Polym. Sci. 138 (16), 50225. 10.1002/app.50225

[B49] Sarfraz R. M. Ahmad M. Mahmood A. Ijaz H. (2018). Development, *in vitro* and *in vivo* evaluation of pH responsive β-CD-comethacrylic acid-crosslinked polymeric microparticulate system for solubility enhancement of rosuvastatin calcium. Polymer-Plastics Technol. Eng. 57 (12), 1175–1187. 10.1080/03602559.2017.1373401

[B50] Sarker S. R. Aoshima Y. Hokama R. Inoue T. Sou K. Takeoka S. (2013). Arginine-based cationic liposomes for efficient *in vitro* plasmid DNA delivery with low cytotoxicity. Int. J. Nanomedicine 8, 1361–1375. 10.2147/ijn.s38903 23630419 PMC3626367

[B51] Sarker S. R. Arai S. Murate M. Takahashi H. Takata M. Kobayashi T. (2012). Evaluation of the influence of ionization states and spacers in the thermotropic phase behaviour of amino acid-based cationic lipids and the transfection efficiency of their assemblies. Int. J. Pharm. 422 (1), 364–373. 10.1016/j.ijpharm.2011.10.044 22079713

[B52] Sarker S. R. Hokama R. Takeoka S. (2014). Intracellular delivery of universal proteins using a lysine headgroup containing cationic liposomes: Deciphering the uptake mechanism. Mol. Pharm. 11 (1), 164–174. 10.1021/mp400363z 24224643

[B53] Sarker S. R. Takeoka S. (2018). Amino acid-based liposomal assemblies: Intracellular plasmid DNA delivery nanoparticles. J. Nanomed 2, 1008–1021. 10.33582/2578-8760/1008

[B54] Sarker S. R. Takikawa M. Takeoka S. (2020). *In vitro* delivery of cell impermeable phallotoxin using cationic liposomes composed of lipids bearing lysine headgroup. ACS Appl. Bio Mater. 3 (4), 2048–2057. 10.1021/acsabm.9b01167 35025326

[B55] Shabir F. Erum A. Tulain U. R. Hussain M. A. Ahmad M. Akhter F. (2017). Preparation and characterization of pH sensitive crosslinked Linseed polysaccharides-co-acrylic acid/methacrylic acid hydrogels for controlled delivery of ketoprofen. Des. Monomers Polym. 20 (1), 485–495. 10.1080/15685551.2017.1368116 29491820 PMC5784885

[B56] Shah S. A. Sohail M. Minhas M. U. Khan S. Hussain Z. Mahmood A. (2019). pH-responsive CAP-co-poly (methacrylic acid)-based hydrogel as an efficient platform for controlled gastrointestinal delivery: fabrication, characterization, *in vitro* and *in vivo* toxicity evaluation. Drug Deliv. Transl. Res. 9 (2), 555–577. 10.1007/s13346-018-0486-8 29450805

[B57] Sharma A. Chopra H. Singh I. Emran T. B. (2022). Physically and chemically crosslinked hydrogels for wound healing applications. Int. J. Surg. 106, 106915. 10.1016/j.ijsu.2022.106915 36115531

[B58] Shawan M. Islam N. Aziz S. Khatun N. Sarker S. R. Hossain M. (2019). Fabrication of xanthan gum: Gelatin (xnt: Gel) hybrid composite hydrogels for evaluating skin wound healing efficacy. Mod. Appl. Sci. 13 (3), 101. 10.5539/mas.v13n3p101

[B59] Singh A. Sharma P. K. Garg V. K. Garg G. (2010). Hydrogels: A review. Int. J. Pharm. Sci. Rev. Res. 4 (2), 97–105.

[B60] Singh A. Sharma P. K. Malviya R. (2011). Release behavior of drugs from various natural gums and polymers. Polim. w Med. 41 (4), 73–80. 22332328

[B61] Subedi M. Bajaj S. Kumar M. S. Mayur Y. (2019). An overview of tramadol and its usage in pain management and future perspective. Biomed. Pharmacother. 111, 443–451. 10.1016/j.biopha.2018.12.085 30594783

[B62] Swami Hulle N. R. Patruni K. Rao P. S. (2014). Rheological properties of aloe vera (A loe barbadensis miller) juice concentrates. J. Food Process Eng. 37 (4), 375–386. 10.1111/jfpe.12093

[B63] Vazzana M. Andreani T. Fangueiro J. Faggio C. Silva C. Santini A. (2015). Tramadol hydrochloride: Pharmacokinetics, pharmacodynamics, adverse side effects, co-administration of drugs and new drug delivery systems. Biomed. Pharmacother. 70, 234–238. 10.1016/j.biopha.2015.01.022 25776506

[B64] Wang Y.-h. Zhong M. Wang L. Liu Y.-l. Wang B. Li Y. (2019). Chelerythrine loaded composite magnetic thermosensitive hydrogels as a novel anticancer drug-delivery system. J. Drug Deliv. Sci. Technol. 54, 101293. 10.1016/j.jddst.2019.101293

